# Interleukin-1 Receptor Blockade Rescues Myocarditis-Associated End-Stage Heart Failure

**DOI:** 10.3389/fimmu.2017.00131

**Published:** 2017-02-09

**Authors:** Giulio Cavalli, Marco Foppoli, Luca Cabrini, Charles A. Dinarello, Moreno Tresoldi, Lorenzo Dagna

**Affiliations:** ^1^Unit of Immunology, Rheumatology, Allergy and Rare Diseases, IRCCS San Raffaele Scientific Institute, Milan, Italy; ^2^Department of Medicine, University of Colorado Denver, Aurora, CO, USA; ^3^Department of Internal Medicine, Radboud University Medical Centre, Nijmegen, Netherlands; ^4^Division of Oncology, IRCCS San Raffaele Scientific Institute, Milan, Italy; ^5^Division of Anesthesia and Intensive Care, IRCCS San Raffaele Scientific Institute, Milan, Italy; ^6^Department of Internal Medicine and Advanced Therapies, IRCCS San Raffaele Scientific Institute, Milan, Italy

**Keywords:** inflammation, inflammasome, heart failure, anakinra, critical care, shock, cytokine

## Abstract

Support measures currently represent the mainstay of treatment for fulminant myocarditis, while effective and safe anti-inflammatory therapies remain an unmet clinical need. However, clinical and experimental evidence indicates that inhibition of the pro-inflammatory cytokine interleukin 1 (IL-1) is effective against both myocardial inflammation and contractile dysfunction. We thus evaluated treatment with the IL-1 receptor antagonist anakinra in a case of heart failure secondary to fulminant myocarditis. A 65-year-old man with T cell lymphoma developed fulminant myocarditis presenting with severe biventricular failure and cardiogenic shock requiring admittance to the intensive care unit and mechanical circulatory and respiratory support. Specifically, acute heart failure and cardiogenic shock were initially treated with non-invasive ventilation and mechanical circulatory support with an intra-aortic balloon pump. Nevertheless, cardiac function deteriorated further, and there were no signs of improvement. Treatment with anakinra, the recombinant form of the naturally occurring IL-1 receptor antagonist, was started at a standard subcutaneous dose of 100 mg/day. We observed a dramatic clinical improvement within 24 h of initiating anakinra. Prompt, progressive amelioration of cardiac function allowed weaning from mechanical circulatory and respiratory support within 72 h of anakinra administration. Recent studies point at inhibition of IL-1 activity as an attractive treatment option for both myocardial inflammation and contractile dysfunction. Furthermore, IL-1 receptor blockade with anakinra is characterized by an extremely rapid onset of action and remarkable safety and may thus be suitable for the treatment of patients critically ill with myocarditis.

## Introduction

A 65-year-old Caucasian man was hospitalized for low-grade fever, fatigue, and unintentional weight loss. He had a history of elevated arterial blood pressure and benign prostatic hyperplasia; family and social history were unremarkable. At admission, vitals were normal. Physical examination revealed a moderately enlarged spleen, a finding confirmed at bedside abdominal ultrasound (bipolar diameter 15 cm). Blood tests showed mild leukocytosis (15,000/mm^3^, neutrophils 88%), anemia (Hb 9.5 g/dL), thrombocytopenia (70,000/mm^3^), high levels of inflammatory markers C-reactive protein (CRP, 104 mg/L, normal values 0–6 mg/L) and ferritin (515 ng/mL, normal values 15–150 ng/mL), and mildly elevated liver transaminases and serum lactate dehydrogenase. A whole-body computed tomography scan revealed confluent areas of reduced tissue density in the liver and spleen, compatible with malignancy, and multiple abdominal lymphadenopathies. In the hypothesis of hematologic neoplasm, a bone marrow biopsy was performed. Histologic examination was eventually diagnostic for anaplastic T cell lymphoma; macrophages phagocytizing hematopoietic cells were concomitantly observed.

Shortly after this procedure, the patient acutely developed fever, hypotension, and dyspnea. Blood pressure dropped to 80/45 mmHg, respiratory rate was 34 breaths per minute, and oxygen saturation was 89% while he was breathing ambient air. Biochemical analyses showed marked leukocytosis with neutrophilia (20,000/mm^3^, neutrophils 90%), elevated CRP (150 mg/L, normal values <6), and troponin T (78 ng/L, normal values <38). Blood and urine cultures were negative, and procalcitonin serum levels were normal. Electrocardiogram showed sinus tachycardia with T wave alterations. A transthoracic echocardiogram (TTE) revealed a severely depressed contractile function in both ventricles, with a left ventricle ejection fraction (LVEF) of 25%. A repeated whole-body computed tomography documented initial pulmonary edema but no remarkable findings in addition to the previously documented liver and spleen abnormalities. Heart catheterization revealed normal coronary anatomy without significant stenosis. Myocardial biopsy was not performed. A comprehensive search for autoantibodies and viral genome testing on blood and sputum were negative.

The patient was admitted to the intensive care unit and received intravenous dobutamine, furosemide, and non-invasive ventilation with continuous positive air pressure. Due to persistent hypotension refractory to positive inotropic agents, urgent positioning of an intra-aortic balloon pump (IABP) was required to maintain the cardiac output. However, during the following day, no signs of myocardial recovery were observed, and the severe biventricular dysfunction further worsened (LVEF < 20%).

## Background

Fulminant myocarditis is an inflammatory disease of the myocardium leading to arrhythmia or rapidly progressive heart failure, often with a fatal outcome. Fulminant myocarditis can be caused by various infectious or exogenous agents, as well as immune-mediated conditions. Regardless of the initiating factor, the pathogenesis is characterized by a deregulated inflammatory response, leading to cardiac damage. This inflammation-mediated myocardial damage leads to contractile dysfunction, clinically manifested with heart failure. Recent studies indicate that the pro-inflammatory cytokine interleukin (IL)-1 is central to the development of cardiac inflammation in the pathogenesis of a broad spectrum of conditions. Mechanistically, during myocardial injury, dying myocardiocytes release various intracellular debris and mediators, including IL-1α, which are sensed by resident and infiltrating myeloid cells (1). Upon sensing of intracellular mediators, the myeloid inflammatory cells activate the “inflammasome,” an intracellular molecular complex responsible for the processing and release of active IL-1β (2–4). The release of IL-1β results in widespread inflammation (5, 6), leading to death of cardiomyocytes, progressive loss of viable contractile tissue, and development of cardiomyopathy and heart failure (7).

Clinical observations confirm this central role of IL-1 in the pathogenesis of cardiac inflammation. In general, myocardial involvement is part of the clinical spectrum of inflammatory (8–10) or autoinflammatory diseases (11, 12), such as adult-onset Still’s disease (AOSD) or systemic onset juvenile idiopathic arthritis (SOJIA), which are characteristically mediated by IL-1. IL-1 receptor blockade is highly effective in these conditions (13), as exemplified by several published cases of myocarditis associated with SOJIA and AOSD and promptly controlled by anakinra (14–17). The clinical picture of this patient, characterized by acute biventricular failure with cardiogenic shock and robust systemic inflammation, was reminiscent of IL-1-mediated autoinflammatory diseases (13). Specifically, the patient had high fever and elevated blood neutrophils but no evidence of infection, non-ischemic acute collapse of cardiac function compatible with myocarditis, and biochemical tests compatible with macrophage activation syndrome (multilinear cytopenia and elevated liver transaminases and lactate dehydrogenase) (18).

Management of fulminant myocarditis remains a clinical challenge, as no specific treatment is available beyond mechanical support (19, 20). We had previously treated a patient with life-threatening viral myocarditis using anakinra, the recombinant form of the naturally occurring IL-1 receptor antagonist (21). That patient had fulminant viral myocarditis requiring venous-arterial extracorporeal membrane oxygenation and mechanical circulatory support with a left-ventricular assist device, and we observed a dramatic clinical response to IL-1 inhibition with anakinra.

## Discussion

The present study reports the dramatic efficacy of IL-1 blockade in a case of fulminant myocarditis. The patient had developed fulminant myocarditis secondary to the immune activation and deregulation induced by an underlying hematologic neoplasm, anaplastic T cell lymphoma, which is frequently associated with rampant, systemic inflammatory activation (22). Clinically, this patient had a progressive collapse of the heart function, despite mechanical circulatory support. Given this life-threatening condition and the absence of established treatment options for fulminant myocarditis beyond mechanical support, we evaluated IL-1 inhibition. Treatment with anakinra was started at a standard subcutaneous dose of 100 mg/day (13, 23). A striking clinical improvement was observed within 24 h of treatment initiation. Clinical response was initially characterized by a swift reduction of fever, neutrophilia (7,000/mm^3^), CRP (10 mg/L, normal values <6), and ferritin (112 ng/mL, normal values 15–150 ng/mL). Over the following 2 days, ECG abnormalities also normalized, as did troponin T levels (33 ng/L, normal values <38), and the LVEF was restored to 50%. After 72 h of daily anakinra, the patient could be weaned from IABP and non-invasive ventilation. Upon clinical stabilization, the patient was transferred to the Oncology Unit. Anakinra was discontinued, and chemotherapy with a CHOEP combination scheme was initiated. Six months after discharge, the patient is continuing to receive treatment with chemotherapy regimens in an outpatient setting. During this time, repeated cardiologic monitoring and TTE re-evaluations confirmed complete recovery of the LVEF (55%).

In addition to experimental evidence, clinical experience with specific IL-1 inhibition provides unambiguous confirmation to the role of IL-1 in human myocardial inflammation and heart failure. For example, a short course of 14 days of anakinra was evaluated in addition to standard of therapy in randomized trials of ST-elevation myocardial infarction (STEMI). Specifically, STEMI patients receiving anakinra had reduced left ventricular remodeling and were protected from the development of heart failure (24). In a different trial evaluating more clinically stable patients after reperfused STEMI, anakinra added to optimal therapy did not result in remarkable benefits on left ventricular remodeling, yet a significant dampening of the systemic inflammatory response confirmed the central relevance of IL-1 to the sterile heart inflammation associated with STEMI (25). In a separate, randomized clinical trial of non-STEMI patients, anakinra treatment was again associated with a dampening of serum inflammatory markers, thus confirming that IL-1 is a driving force of cardiac and systemic inflammation (26).

Given the central role of IL-1 in cardiac inflammation, it is not unexpected that treatment with anakinra would curb inflammation and afford significant clinical benefits. However, the beneficial effects of IL-1 inhibition on cardiac function extend beyond dampening of inflammation and include favorable effects on cardiac contractility. Specifically, *ex vivo* studies with human atrial heart strips revealed that IL-1 suppresses contractile force, even at picomolar concentrations (27). Consistently, administration of anakinra to patients with refractory heart failure improved exercise tolerance and was associated with a corresponding decrease in major pro-inflammatory mediators CRP, IL-1β, and IL-6 (28). In a separate double-blind, placebo-controlled trial, IL-1 inhibition with anakinra 100 mg daily was effective also in the management of diastolic heart failure (29). This direct beneficial effect of IL-1 blockade on contractile function likely explains the near-immediate clinical improvement in the case described in the present report. This observation is not unprecedented, as a single dose of anakinra increased cardiac contractility in patients with rheumatoid arthritis within hours of administration (30).

Anakinra blocks the biologic activity of both IL-1α and IL-1β by competitively inhibiting binding to the interleukin-1 type I receptor, which is constitutively expressed in most tissues and organs. IL-1α and IL-1β levels are elevated in response to inflammatory stimuli. While levels of endogenous IL-1Ra are often too low to contain an overwhelming IL-1-mediated inflammatory reaction, exogenous administration with anakinra suppresses the pathological process. It remains unresolved how much of the efficacy of anakinra is attributable to either IL-1α or IL-1β inhibition. In this study, we did not determine IL-1α or IL-1β levels. However, cytokine levels in patients with systemic or local inflammation do not reveal causation, regardless of the correlation. Even in patients with established IL-1-mediated diseases such as AOSD or SOJIA, the levels are often below detection, but the rapid response to anakinra provides clear evidence for causation. Similarly, a rapid response to anakinra indicates a causative role for IL-1-mediated inflammation in myocarditis.

Support measures currently represent the mainstay of treatment for fulminant myocarditis (19, 20, 31). In the case described in the present report, patient’s heart function had swiftly deteriorated and was dependent on mechanical circulatory support with IABP; nevertheless, IL-1 inhibition near immediately restored cardiac contractility. Caution is advisable in ascribing the recovery of patients with fulminant myocarditis to specific therapeutic interventions, as spontaneous recovery may occasionally occur during mechanical circulatory support. Specifically, patients presenting with fulminant myocarditis, although more severely ill, may be more likely to fully recover than those affected by chronic myocarditis, provided they survive the acute phase of the disease (32). However, several reasons point at a causative role for IL-1 inhibition in the full recovery of cardiac function herein described. First, we observed a close and striking temporal relation between treatment administration and recovery, which halted a downward spiraling trend. In addition, the concomitant dampening of systemic inflammation cannot be attributed to mechanical circulatory support. Finally, a rationale for IL-1 inhibition in the treatment of heart inflammation is evident well beyond our clinical experience in the intensive care unit and is supported by sound clinical and experimental evidence (33). It remains instead to be determined whether the systemic anti-inflammatory effects of treatment may have also contributed, at least in part, to promoting cardiac recovery.

Important for the administration to the critically ill, anakinra has a remarkable record of safety, as a short half-life of 6 h allows prompt discontinuation [reviewed in Ref. (13)]. Adverse events include injection site reactions and mild-to-moderate neutropenia, which is rapidly reversible upon cessation of treatment.

## Conclusive Remarks

Regardless of the triggering agent, the inflammatory response in myocarditis rapidly escalates into an auto-inflammatory cycle. Mechanistically, intracellular contents released from dying myocardiocytes trigger the inflammasome activation and the release of IL-1 from neighboring cells, leading to rampant inflammation and tissue damage (2). Prompt pharmacologic inhibition of IL-1 can arrest the progression of uncontrolled inflammation, thus preventing extensive damage and restoring cardiac function. Given the dual efficacy against myocardial inflammation and contractile dysfunction, IL-1 inhibition represents an attractive treatment option for conditions characterized by inflammatory heart failure.

Interleukin-1 receptor blockade with anakinra is characterized by an extremely rapid onset of action and by satisfying safety, thus being suitable for the treatment of life-threatening conditions. Indeed, intravenously administered high-dose anakinra was previously evaluated in the treatment of septic shock in three controlled trials taking place in the 1990s (34–36). However, dated and loose criteria for septic shock lead to the enrollment of highly heterogeneous patients, and anakinra did not reach statistical significance for reducing all-cause 28-day mortality in this setting. Nevertheless, better therapeutic performance was observed in patients with the highest risk of death, bacteremia, or elevated serum ferritin (36, 37).

Further studies of IL-1 blockade in myocarditis are needed to validate use in critically ill patients. Specifically, prospective evaluation of a 2-week treatment course with anakinra 100 mg daily in addition to conventional life support measures may unequivocally assess the impact on acute and mid-term LVEF changes. Additional outcome measures of interest include survival, requirements for and doses of conventional pharmacologic therapy, assessments of symptoms, and arrhythmias.

## Author Contributions

All authors contributed substantially to this work, have approved the manuscript, and agreed with its submission.

## Conflict of Interest Statement

The authors declare that the research was conducted in the absence of any commercial or financial relationships that could be construed as a potential conflict of interest. The reviewer JA and handling editor declared their shared affiliation, and the handling editor states that the process nevertheless met the standards of a fair and objective review.
